# Loot

**Published:** 1871-08

**Authors:** 


### Loot.

***Cundurango.***

"Cundurango," which we had the honor of introducing to the notice of our readers a few weeks ago, is fairly started on its therapeutical travels under the patronage of the State Department, and for some time to come will furnish a theme for eulogistic admiration in daily newspapers, albeit its miraculous attributes have not, thus far, excited the enthusiasm of the medical press. We are, however, glad to announce that one contribution of a scientific character has been made to its history by Professor Antisell, to whom a specimen was forwarded for examination, the result of which is recorded in the *National Medical Journal.* The absence of root or leaves in the samples sent prevented the determination of the botanical relations of the plant, and the physical description is limited to the following particulars: The stem is "woody, shrubby, and covered by a greenish or ash-gray bark, the former tint being due to a coating of lichens on the surface." The woody fibre is straw-colored and brittle, very slightly aromatic and bitter in taste. The bark is slightly corrugated, easily separable from the stem, having an aromatic, bitter flavor like that of cascarilla, and divisible into three layers; the inner consisting of reticular woody fibre, with starch granules and particles of resin; the middle of fibre and dotted ducts also containing resinous particles; and the outer of bark-cells containing tannic acid and coloring matters. The chemical analysis of the bark showed:—

| | |
|---|---|
| Moisture, - - - - - - - - - - - - - - | 8 |
| Mineral salts, - - - - - - - - - - - - - | 12 |
| Vegetable matters, - - - - - - - - - - - - - - | 80 |
| | 100 |

Further examination reduced these vegetable matters to:—

| | |
|---|---|
| Fatty matter, soluble in ether and partially in strong alcohol, - | .7 |
| Yellow resin, soluble in alcohol, - - - - - - - - | 2.7 |
| Starch, gum, and glucose, - - - - - - - - - - | .5 |
| Tannin, yellow and brown coloring matter and extractive, - | 12.6 |
| Cellulose, lignin, etc., - - - - - - - - - - | 63.5 |
| | 80. |

No volatile oil or acid, no alkaloid or active principle, could be obtained. Dr. Antisell classes cundurango among the aromatic bitters, and thinks that whatever medicinal properties it may possess must reside either in the yellow resin or in the extractive, the former of which is soluble in alcohol, the latter in water.

Although, of course, all therapeutical questions must be practically settled by clinical rather than chemical tests, the above showing is not calculated to increase our faith in the triune specificity of cundurango for "cancers, ulcerous affections, and venereal diseases." So many cures for carcinoma have successively been extolled and failed—arsenical paste, acetic acid, gastric juice, carbolic acid, electrolysis, and a host of others—that we fear this dread malady must long remain an *opprobrium medicorum.* It is beyond question that, in some rare cases, spontaneous recovery from cancer occurs; but in such instances the treatment cannot be accepted as influencing the result, and we confess our very strong doubt whether a specific remedy will ever be discovered—if, indeed, there exist a specific for any disease whatsoever. —*Med. Gazette.*

### *Treatment of Hæmatemesis by Transfusion.*

Dr. Carl Michel reports an interesting case of hæmatemesis in which transfusion was successfully employed. The patient was 63 years of age, robust, but had suffered for a few weeks previously from loss of appetite and some eructations. On the 15th of May he became listless and unable to do his work, and went to bed and took a dose of salts, and for three or four days suffered from diarrhœa. The motions were black. He became very weak, cold sweats appearing on his face. On the 19th, M. Michel saw him. He was lying on his back, very debilitated and anæmic, with strong abdominal pulsation, and pain on the pressure of the abdomen. Whilst examining him the pupil contracted, several drops appeared on the forehead, and the whole body began to tremble. This lasted for a minute. On placing him upon a night stool, three black lumps, the size of a hen's egg, were passed, with a pint of clear blood. The patient then fainted. The diagnosis was then made as being either a rupture of a degenerated atheromatous artery or as chronic ulcer of the stomach. He was ordered ice internally and externally, acetate of lead and opium, and perfect rest. On the following day there was improvement, and in exchange for lead and opium, diluted sulphuric acid was ordered in small doses every two hours. On the 21st, vomiting of blood occurred, and all the symptoms became worse. Infusion of digitalis and nitrate of potash was ordered, and subsequently, as no improvement followed, tinct. ferri acetat. The diet was milk and cold beef tea. On the 22d, he was insensible for three-quarters of an hour, and all his symptoms betokened the near approach of death. Transfusion was determined upon, and performed on the 24th with a simple glass syringe, without the help of a skilled assistant. The blood was taken from the arm of the son, and received to the extent of four ounces into a vessel floating on warm water; here it was whipped and immediately injected into the cephalic vein, which had been previously laid bare and surrounded with a ligature, by which it was lifted out of its bed. The blood was slowly injected, care being taken that no air entered. No sensation of dyspnœa was experienced. During the injection, and after it, he said he felt easier. An hour afterwards he had a rigor, but no fainting or convulsions recurred. He was directed to continue the use of the iron, and to take cold broth with yolk of eggs. On the 26th, he was much better, but some tenesmus occurring, a starch clyster was ordered with six drops of tincture of opium, and from this time he steadily regained his health. Upon the whole it did not appear that more than 1½ oz. were really injected.—(*Berliner Klinische Wochenschrift*, No. 49, 1870.)—*Practitioner.*

***Treatment of Sciatica.***

Dr. J. Ernest Meiere, M.D., writes to the *Medical Record:*—The following treatment which I have adopted in three cases of sciatica of several years' standing, with the most gratifying results, I publish to the profession, believing by its strict observance we have the means of controlling and of eradicating this disease.

I shall give the history of its treatment in one case, it being the most severe.

Thomas Browning, aged 45, a farmer of Western Maryland, presented himself at my office for treatment, having for the eighteen months previous suffered from sciatica. The entire right lower limb was *atrophied*, and he said he could *span it at the largest part of the thigh with one hand.* He was entirely incapacitated from attending to the duties of his farm, and could barely "hobble" about with the aid of a stick. He was very much discouraged, having previously to my seeing him been under the treatment of several physicians "without much benefit." On my giving him assurance that I could benefit him, he placed himself under my care on the 4th of April, 1870, and continued under my treatment, at intervals, until July, when I saw him professionally for the last time. The treatment was as follows:

He was cupped along the course of the sciatic nerve to the ankle, taking about eight ounces of blood. He was then ordered:

℞ Ol. tiglii gtt. iii.
Fiat in pil. No. 3. Sig., one each night.

Should the pills operate too frequently, their action can be easily checked with tinct. opii.

The following mixture was then given him, which I am confident has a very great controlling influence on the disease.

* ℞ Quiniæ sulph. ℈iss. Vini colch. sem. f℥i.

Potassii iodidi, ℥ss. Aquæ dest. ℥vi.

Sig., teaspoonful three times daily.

The cupping was repeated on the 8th and 23d of the same month, taking about the same quantity of blood as at the first operation. The mixture was continued at intervals up to July, but I should advise that there be no interruption in its administration until the termination of the treatment.

The limb should be subjected to a cold water douche, morning and night, for five or ten minutes, for several months thereafter, and strict attention be paid to the bowels.

### *The New York Ear Dispensary.*

A much-needed charity, whose object is indicated by its title, has been recently opened at No. 69 West 35th Street, near 6th Avenue. It was incorporated in April last, and is under the control of a board of twelve trustees, who have appointed as attending surgeons Drs. Geo. B. Pomeroy and Samuel Sexton.

The importance of the ear as a field of investigation is being generally recognized, and its study is taking rank with the other specialties of the day.

* To prevent flocculent solution, the quinine and iodide of potassa should be dissolved in different vessels, or else a precipitate of the insoluble iodide of quinine will be thrown down, and the medicine be of no effect.

